# Altmetric attention and citation performance in sport science: a cross-sectional analysis of 30 top-ranked journals

**DOI:** 10.3389/fspor.2025.1710760

**Published:** 2026-01-12

**Authors:** Yanbing Zhou, Ziwei Luo

**Affiliations:** Department of Kinesiology and Health Education, The University of Texas at Austin, Austin, TX, United States

**Keywords:** altmetric score, citation count, exercise, health, journal impact factor, sport

## Abstract

**Background:**

Disconnection between efficacy and implementation remained in sport and exercise science, making it vital to understand the relationship between research quality and public attention. Citation score (CS) and Journal impact factor (JIF) are the main metrics of research quality. Alternatively, Altmetric score (AS) tracks article dissemination on social media to reflect public attention. This study aimed to explore the CS-AS correlations on levels of article types and journals, and predictors of citation performance.

**Methods:**

Web of Science Core Collection was systematically searched for research and review studies published in 2022 in the top-ranked 30 journals under the sport science category of Journal Citation Reports™. Publications from 2022 were selected to accumulate citations while preserving contemporary representativeness. CS and AS scores were retrieved from the website Dimensions (https://app.dimensions.ai/discover/publication).

**Results:**

A total of 5,106 articles were retrieved with a median CS and AS of 10 and 6, respectively. A significant CS-AS correlation was observed (*ρ* = 0.3481, *p* < 0.0001). The correlation strength of the review study (*ρ* = 0.4047, *p* < 0.0001) was significantly higher (*p* = 0.001) than that of research articles (*ρ* = 0.2975, *p* < 0.0001). Significant CS-AS correlations were found in 25 out of 30 journals (*ρ* ranged from 0.7084 to 0.1238, *p* ranged from 0.05 to <0.0001). AS and JIF were significant predictors of CS, with the strength of AS (*β* = 0.2060, *p* < 0.001) higher than JIF (*β* = 0.0720, *p* < 0.001).

**Conclusion:**

In the 30 top-ranked sport science journals, media attention was correlated with citation performance, with the strength higher in reviews than in research articles. The significant correlations showed up in 25 out of 30 included journals with different strengths. Social media attention can be a more powerful predictor than journal prestige in sport science, with a moderate predictive influence.

## 1 Introduction

The volume of research in sports and exercise science has grown exponentially over the past decades (1). However, there is still a distinct disconnect between efficacy and real-world implementation (2). Despite the longstanding establishment of the physical activity guideline (3), the global obesity rate continues to rise (4). Barely half of the adults in the U.S. could accumulate at least 150 min/week of moderate-to-vigorous physical activity (5). Only 4% can identify the recommendations in both aerobic and muscular exercises (6). Similarly, multiple guidelines of sport-related injuries have been published; however, sport teams, including team physicians, have yet to consistently implement this knowledge and the best practice (7, 8). These disconnects raise an important question about how sport and exercise science research is communicated, perceived, and utilized by the sports team and the general public.

To address this issue, it is crucial to understand the relationship between scientific research and public attention. Citation score (CS) and journal impact factor (JIF) are two critical metrics to measure the quality and value of a research article (9, 10). CS is determined by how often an article is cited by other articles. The JIF (Clarivate™, JISC Services Ltd., Bristol, UK) is defined as the average number of citations for an article in a particular journal within two years after publication. While CS and JIF take time to accumulate, alternatively, Altmetric Score (AS) serves as a real-time metric to track public attention of a particular article on social media. The AS is derived from an automated algorithm and weighted to reflect the reach and credibility of each platform, such as blogs, Wikipedia, and social media posts (e.g., X, Facebook, blogs, Mendeley, and news outlets). AS is computed in near-real time but may continue to evolve months after publication, depending on engagement trends.

Despite that, in the previous study (11), the relation between CS and AS was analyzed, showing high heterogeneity, reducing the confidence and credibility in the pooled estimate. Therefore, this cross-sectional study aimed to explore the relationship between CS and AS on different layers, including the factors that could predict future citation performance. To our knowledge, this is the first study to perform stratified analyses on main article types and individual journals in sport and exercise science.

## 2 Materials and methods

### 2.1 Searching strategy

The Clarivate™ Journal Citation Reports™ (JCR) was utilized to determine the top journals. The JIF values were retrieved, and the top 30 journals under the category of Sport Science with the highest JIF were selected for a systematic search.

The Web of Science (WOS) Core Collection was utilized to perform the systematic search using advanced search strings {[SO = (Journal names)] AND DOP = (2022-01-01/2022-12-31)}. The publication time was restricted to the year of 2022, to allow a 2.5-period for the CS to accumulate (12). The search was performed on July 26th, 2025. The searching process was performed by two independent researchers (Y., Z., and Z., L.). Each reviewer was blinded to the other's results until the final merging step. Disagreement was addressed through discussion if needed.

### 2.2 Inclusion/exclusion criteria

Journals that focused on sports, exercise, clinical health, health promotion, and education would fit the inclusion criteria. The research article and review articles were included for analysis. Journals that concentrated on management (e.g., *Journal of Sport Management*) were excluded. Since the stratified analysis of main article types was part of the purpose of this study, journals that only accepted and published review articles (e.g., *Exercise and Sport Sciences Reviews*, *Exercise Immunology Review*), and Journals that only focused on a specific subfield (e.g., *Science and Medicine in Football*) were excluded to generate extended results. Article types other than research articles and review articles, such as comments, editorials, letters, and corrections, were excluded. Articles that were published outside the time frame of 2022 were excluded.

### 2.3 Data extraction

The search results from WOS Core Collection were input into Microsoft Excel (Version 2306, Microsoft® Corporation, USA, 2024) and EndNote (EndNote 21, Clarivate, USA, 2024) for literature management.

The Dimensions website (https://app.dimensions.ai/discover/publication) was utilized to retrieve the up-to-date CS and AS values. The DOI numbers extracted from the WOS Core Collection were applied to the website for data retrieval. All independent variables, including AS and JIF, were defined *a priori*. The extracted data of CS and AS were collected by two independent researchers (Y., Z., and Z., L.). The extraction process was blinded from each other till they ended. Disagreement was addressed through discussion if needed.

### 2.4 Statistical analysis

The correlations between CS and AS were the primary outcome at the level of article type (research/review), individual journal information, and the Citation-Altmetric overall correlation. The regression analysis was the second outcome to determine if AS and JIF could predict citation performance.

Descriptive analysis was presented with median and interquartile range (IQR), due to the non-normal distribution of the datasets. Correlation was tested using Spearman's rank-order correlation analyses, presented in the form of Spearman's *ρ*, 95% confidence interval (CI), and included sample size (*n*). Multiple testing corrections (Bonferroni) were applied to control for the increased risk of Type I error. The Fisher's r-to-z transformation was performed to compare the correlations from two independent samples (CS and AS). The transformed z-scores were tested for detecting significant differences. Multiple Linear Regression was performed for factor prediction.

The CS and AS scores were log-transformed by equations to reduce the skew and transform them to a normal distribution. Model fit was evaluated by *R*^2^, adjusted *R*^2^, and the lack-of-fit test. Regression results were shown by β estimate, standard error (SE), and *t* ratio. Regression coefficients (β) represent the expected change in the log-transformed citation score for a one-unit increase in the log-transformed Altmetric Score or JIF. Thus, *β* values are interpreted on the transformed scale and reflect proportional rather than absolute changes. A significance level of *α* = 0.05 was used for all statistical tests. Data were shown as median and interquartile range. Correlation strength was interpreted by Spearman's *ρ* as negligible [0.00–0.09], small [0.10–0.39], moderate [0.40–0.69], strong [0.70–0.89], or very strong [0.90–1.00] (13). Analyses were performed using GraphPad Prism (version 10.0.3; GraphPad Software, San Diego, CA, USA, 2023), Psychometrica (14) (https://www.psychometrica.de; accessed July 27, 2025), and JMP Pro (version 18.0; SAS Institute Inc., Cary, NC, USA, 2025).

## 3 Results

### 3.1 Overall correlations

After the systematic search, a total of 5,106 studies from the top 30 journals with the highest IF were included and analyzed. The information on journals and studies output, CS, and AS was shown in Table 1.

**Table 1:** Characteristics of journal outputs, citation scores, and altmetric scores of the 30 top-ranking journals in sport and exercise science.

Journals	Study outputs	Citation scores		Altmetric scores		JCR IF (2024)	JCR quartile
		Median	IQR	Median	IQR		
British Journal of Sports Medicine	127	25	27	74	238	16.2	Q1
Journal of Sport and Health Science	70	28	42.75	9	27	10.3	Q1
Sports Medicine	138	30	33.25	34.5	79	9.4	Q1
Sports Medicine-Open	145	15	19	10	21	5.9	Q1
Journal of Orthopaedic & Sports Physical Therapy	63	14	18	27	29	5.8	Q1
Arthroscopy-The Journal of Arthroscopic and Related Surgery	251	12	13	5	6	5.4	Q1
Biology of Sport	114	12	13	5	8	5	Q1
Knee Surgery Sports Traumatology Arthroscopy	428	10.5	12	1	3	5	Q1
American Journal of Sports Medicine	373	11	13.5	3	7	4.5	Q1
International Journal of Sports Physiology and Performance	219	7	9	8	11	4.3	Q1
Medicine & Science in Sports & Exercise	232	10	12	9	21	3.9	Q1
Journal of The International Society of Sports Nutrition	38	8	9.5	12	22.5	3.9	Q1
Scandinavian Journal of Medicine & Science in Sports	142	9.5	10	6	18.25	3.8	Q1
Archives of Physical Medicine and Rehabilitation	265	8	11	4	6	3.7	Q1
Performance Enhancement & Health	21	10	11.5	6	6	3.7	Q1
Journal of Exercise Science & Fitness	68	9	12.75	3	7	3.5	Q1
Journal of Science and Medicine in Sport	139	9	10	9	15	3.4	Q1
Journal of Applied Physiology	248	8	10	6.5	12	3.3	Q1
Journal of Joint Disorders & Orthopaedic Sports Medicine	40	6.5	10.75	0	1	3.3	Q1
Psychology of Sport and Exercise	171	9	10	3	9	3.3	Q1
BMJ Open Sport & Exercise Medicine	97	7	9	10	17	3.2	Q1
Journal of Applied Sport Psychology	41	8	8.5	4	10	3.2	Q1
Qualitative Research in Sport Exercise and Health	46	7.5	10	3.5	13	3.2	Q1
Journal of Strength and Conditioning Research	498	9	12	8	12	3	Q1
European Journal of Sport Science	164	7	6	5	9.75	3	Q1
Strength and Conditioning Journal	59	5	7	5	15	3	Q1
Journal of Shoulder and Elbow Surgery	386	9	11	1	3	2.9	Q1
Sports	206	7	8	2	5.25	2.9	Q1
Journal of Athletic Training	104	7	11	8.5	15.75	2.8	Q1
BMC Sports Science Medicine and Rehabilitation	213	7	8	1	4	2.8	Q1

IQR, interquartile range; JCR, journal citation reports; IF, impact factor.

Among them, 4,367 research articles (85.5%) and 739 review articles (14.5%) were included. Their study outputs ranged from 21 to 498, with a CS of 10 (6–18) and an AS of 6 (2–15). The highest CS and AS were observed from the *British Journal of Sports Medicine*, with a CS of 25 (27) and an AS of 74 (238). The IFs of the included journals were between 16.2 and 2.8. All the included journals were categorized in JCR Quartile 1. All reported correlations met the significance threshold of *α* = 0.00167 after Bonferroni correction.

### 3.2 Correlations between article type differences

**Figure 1:**
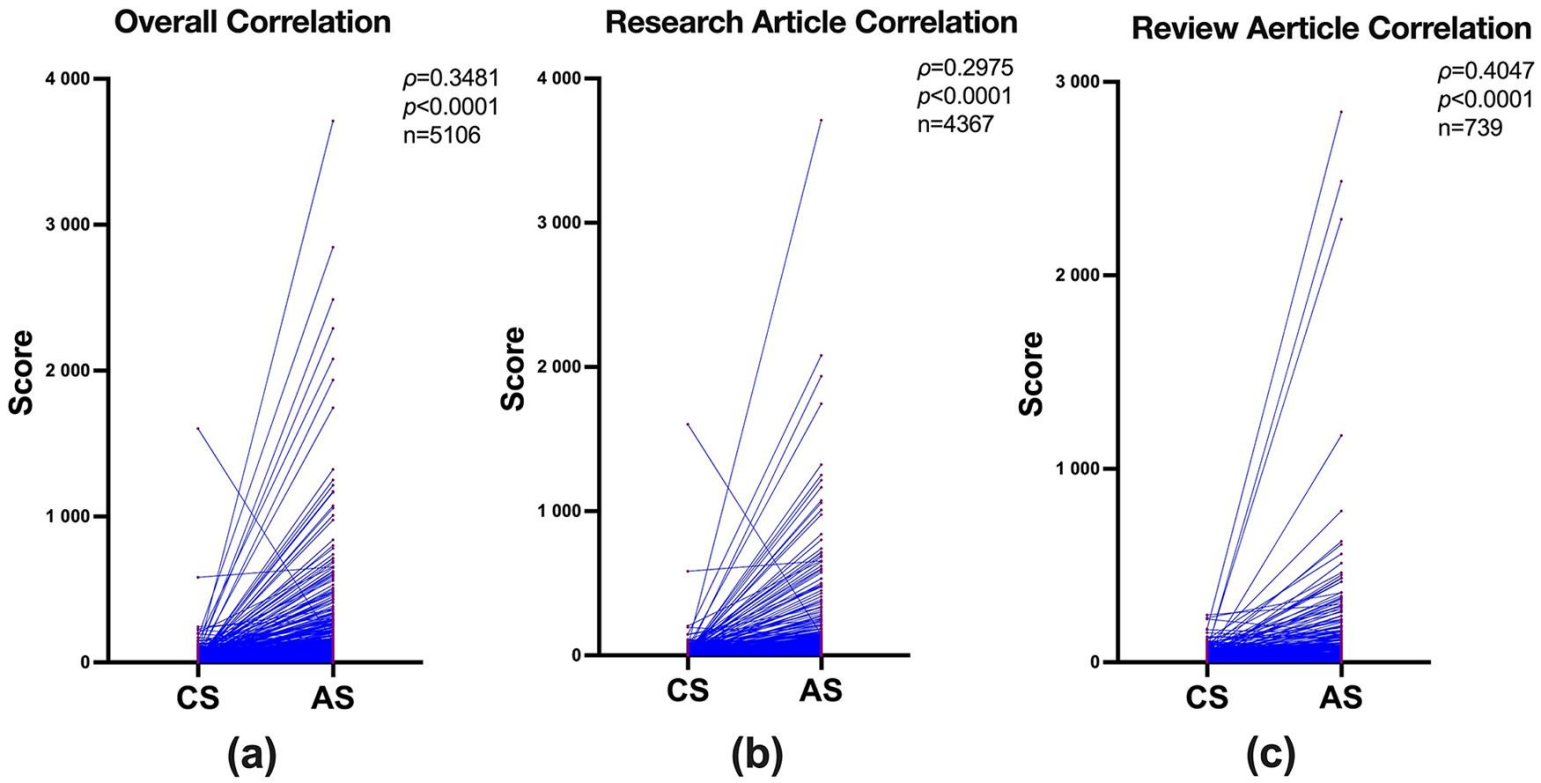
Overall and subgroup correlations between citation scores (CS) and altmetric scores (AS) of the 30 top-ranking journals in sport and exercise science. Paired comparisons of CS and AS across **(a)** research and review articles combined, **(b)** research articles only, and **(c)** review articles only. All correlations were statistically significant (*p* < 0.0001).

As shown in Figure 1, a small but significant correlation (*ρ* = 0.3481, 95% CI 0.3230–0.3726, *p* < 0.0001, *n* = 5,106) between CS and AS was observed in all research and review articles combined (Figure 1a). After subgrouping the article type, a small and a moderate correlation was found in research articles (*ρ* = 0.2975, 95% CI 0.2694–0.3251, *p* < 0.0001, *n* = 4,367) (Figure 1b) and in review articles (*ρ*=0.4047, 95% CI 0.3407–0.4650, *p* < 0.0001, *n* = 739) (Figure 1c).

Despite the magnitude of the overall correlation in Figure 1a being small (*ρ* = 0.3481), this level of association indicates that articles receiving greater online attention tend to receive more citations. The moderate correlation observed for review articles (*ρ* = 0.40) in Figure 1c suggests that public/media attention aligns more strongly with later citation performance for this article type compared to research articles (Figure 1b), in which the association remained small but significant.

**Figure 2:**
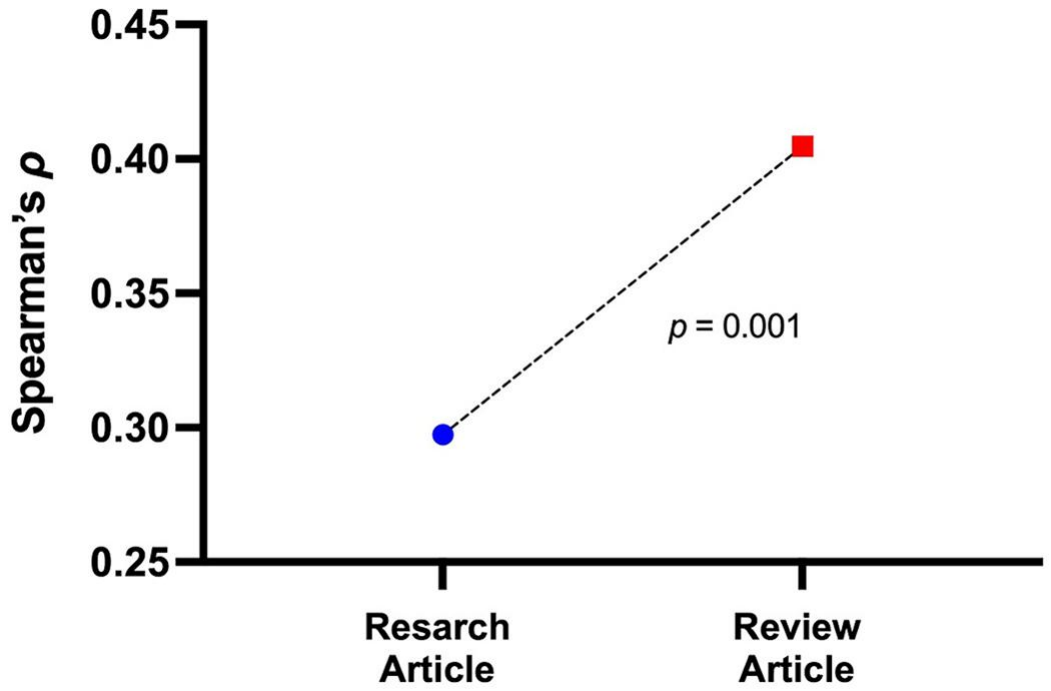
Comparison of citation-altmetrics correlation strength (Spearman's *ρ*) between research and review articles in the 30 top-ranking journals in sport and exercise science.

The correlation of CS and AS between research articles and review articles was demonstrated in Figure 2. The difference in correlation strength was significant (*Z* = −3.074, *p* = 0.001). The review articles tended to show a tighter alignment between citation impact and public/media attention. Both the research study and review study correlations exceeded the Bonferroni-corrected significance threshold (*α* = 0.00167).

### 3.3 Correlations between journal differences

**Figure 3:**
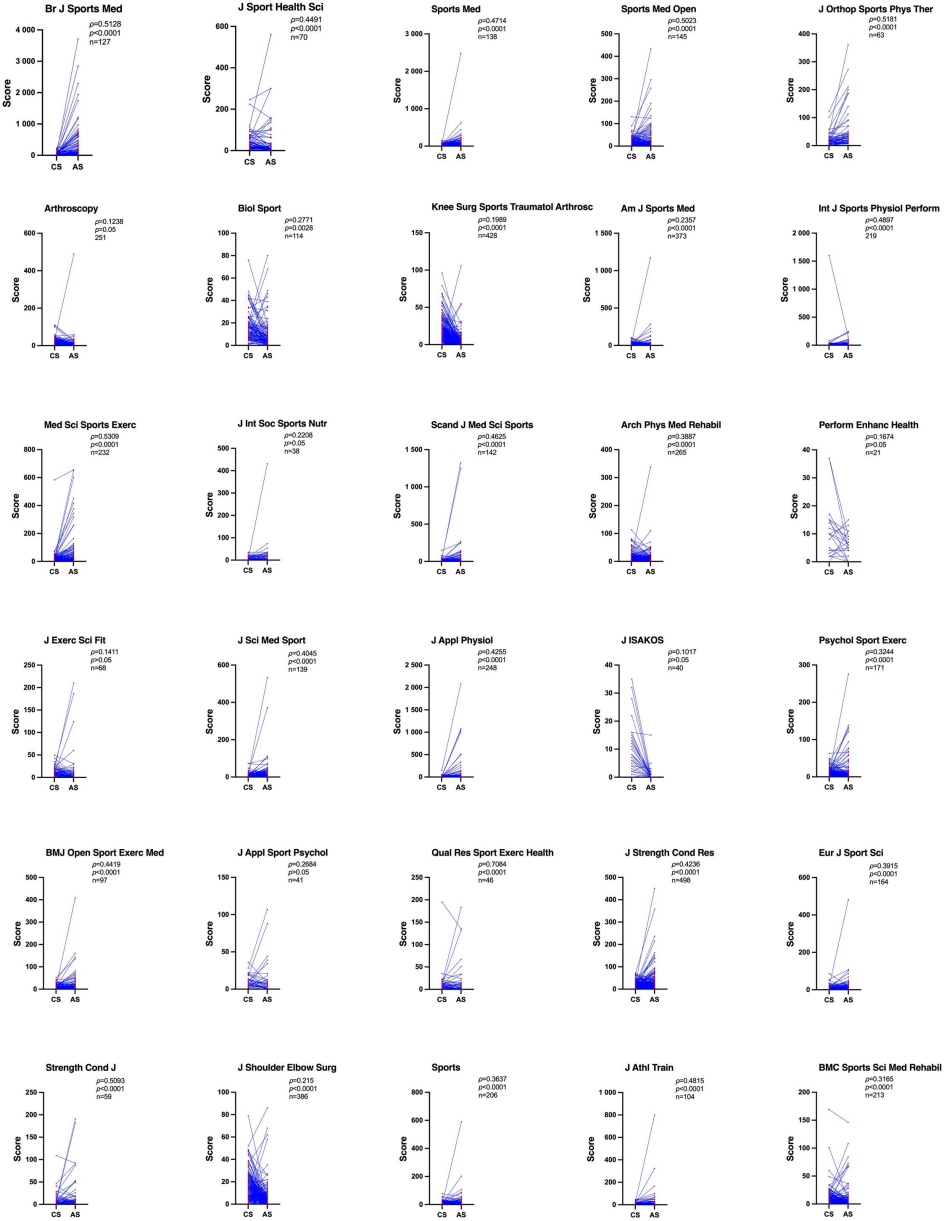
Journal-level correlations between citation scores (CS) and altmetric scores (AS) of the 30 top-ranking journals in sport and exercise science.

The correlations between CS and AS of the 30 top-ranking journals are individually shown in Figure 3. The strongest correlations were observed in *Qualitative Research in Sport Exercise and Health* (*ρ* = 0.7084, 95% CI 0.5200–0.8311, *p* < 0.0001, *n* = 46), indicating a strong and consistent alignment between citation impact and public/media attention in these journals.

Moderate and significant correlations were shown in *Journal of Science and Medicine in Sport* (*ρ* = 0.4045, 95% CI 0.2506–0.5385, *p* < 0.0001, *n* = 139), *International Journal of Sports Physiology and Performance* (*ρ* = 0.4897, 95% CI 0.3786–0.5870, *p* < 0.0001, *n* = 219), *Journal of Athletic Training* (*ρ* = 0.4815, 95% CI 0.3132–0.6204, *p* < 0.0001, *n* = 104), *Sports Medicine* (*ρ* = 0.4714, 95% CI 0.3259–0.5951, *p* < 0.0001, *n* = 138), *Scandinavian Journal of Medicine & Science in Sports* (*ρ* = 0.4625, 95% CI 0.2327–0.6231, *p* < 0.0001, *n* = 70), *Journal of Sport and Health Science* (*ρ* = 0.4491, 95% CI 0.2327–0.6231, *p* < 0.0001, *n* = 70), *BMJ Open Sport & Exercise Medicine* (*ρ* = 0.4419, 95% CI 0.2603–0.5933, *p* < 0.0001, *n* = 97), *Journal of Applied Physiology* (*ρ* = 0.4255, 95% CI 0.3144–0.5251, *p* < 0.0001, *n* = 248), *Journal of Strength and Conditioning Research* (*ρ* = 0.4236, 95% CI 0.3464–0.4950, *p* < 0.0001, *n* = 498). Journals with moderate-to-strong correlations (*ρ* ≥ 0.40) demonstrated a clearer pattern in which higher AS were more reliably associated with higher citation performance, indicating more effective translation of online visibility into scholarly impact.

Small but significant correlations were shown in the following journals: *European Journal of Sport Science* (*ρ* = 0.3915, 95% CI 0.2492–0.5173, *p* < 0.0001, *n* = 164), *Archives of Physical Medicine and Rehabilitation* (*ρ* = 0.3887, 95% CI 0.2780–0.4891, *p* < 0.0001, *n* = 265), *Sports* (*ρ* = 0.3637, 95% CI 0.2350–0.4798, *p* < 0.0001, *n* = 206), *Psychology of Sport and Exercise* (*ρ* = 0.3244, 95% CI 0.1790–0.4560, *p* < 0.0001, *n* = 171), *BMC Sports Science Medicine and Rehabilitation* (*ρ* = 0.3165, 95% CI 0.1863–0.4358, *p* < 0.0001, *n* = 213), *Biology of Sport* (*ρ* = 0.2771, 95% CI 0.0927–0.4431, *p* < 0.0001, *n* = 114), *American Journal of Sports Medicine* (*ρ* = 0.2357, 95% CI 0.1345–0.3321, *p* < 0.0001, *n* = 373), *Journal of The International Society of Sports Nutrition* (*ρ* = 0.2208, 95% CI −0.1161–0.5121, *p* > 0.05, *n* = 38), *Journal of Shoulder and Elbow Surgery* (*ρ* = 0.215, 95% CI 0.1148–0.3109, *p* < 0.0001, *n* = 386), *Knee Surgery Sports Traumatology Arthroscopy* (*ρ* = 0.1989, 95% CI 0.1033–0.2908, *p* < 0.0001, *n* = 428), *Arthroscopy-The Journal of Arthroscopic and Related Surgery* (*ρ* = 0.1238, 95% CI −0.0034–0.2473, *p* = 0.05, *n* = 251).

In *Journal of Joint Disorders & Orthopaedic Sports Medicine* (*Journal of ISAKOS*) (*ρ* = 0.1017, 95% CI −0.2257–0.4085, *p* > 0.05, *n* = 40), *Journal of Exercise Science & Fitness* (*ρ* = 0.1411, 95% CI −0.1079–0.3733, *p* > 0.05, *n* = 68), *Performance Enhancement & Health* (*ρ* = 0.1674, 95% CI −0.2974–0.5680, *p* > 0.05, *n* = 21), *Journal of Applied Sport Psychology* (*ρ* = 0.2684, 95% CI −0.05221–0.5388, *p* = 0.09, *n* = 41), the non-significant correlations were observed.

**Figure 4:**
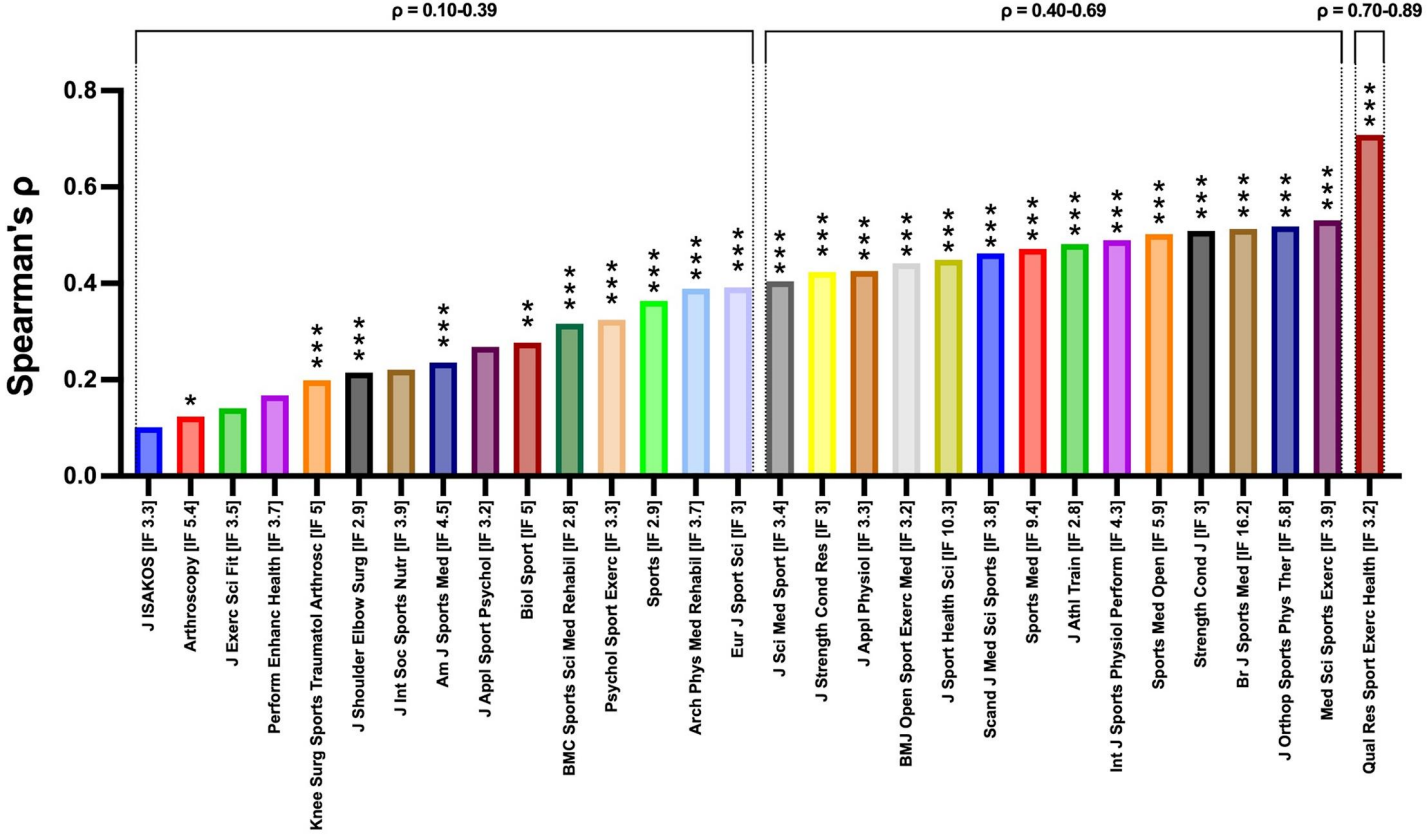
Ranked strengths of citation-altmetric correlations (Spearman's *ρ*) across 30 JCR Q1 sport and exercise science journals. *** *p* < 0.0001, ** *p* < 0.01, * *p* < 0.05. JCR Q1, Journal Citation Reports Quartile 1.

Since most *p*-values in the correlation analyses were <0.0001, they fell below the Bonferroni-corrected significance threshold (*α* = 0.05/30 = 0.00167), indicating the results remained robust to multiple testing. The aforementioned Spearman's *ρ* values were ranked from the lowest to the highest, shown in Figure 4.

### 3.4 Predictors of citation score

**Figure 5:**
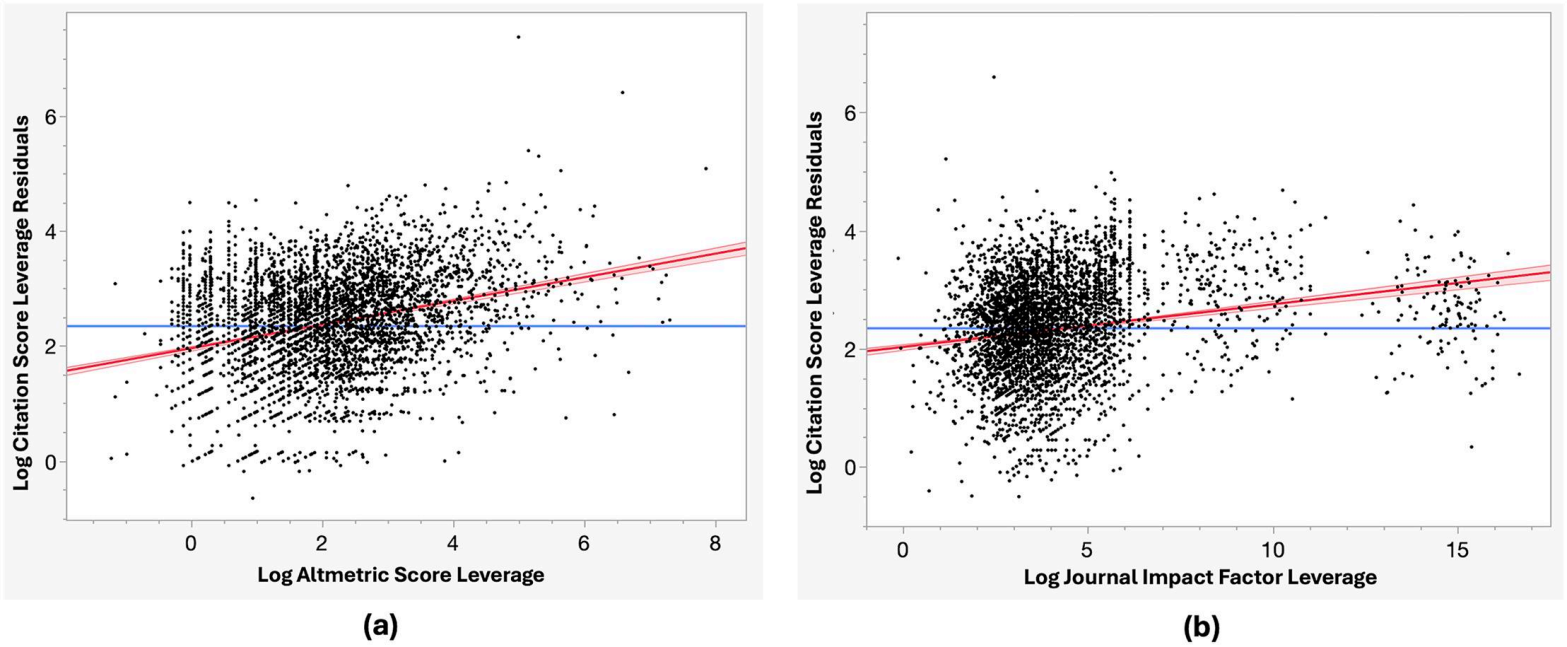
Log-transformed multiple linear regression: predictive influence of altmetric score and journal impact factor on citation score of the 30 top-ranking journals in sport and exercise science. Scatterplots of log-transformed leverage residuals of citation scores as a function of **(a)** Altmetric Score leverage and **(b)** Journal Impact Factor leverage.

The results of the leverage analysis were shown in Figure 5. This model was tested statistically significant: *F*(*k*, *n* - *k* - 1) = *F*(2, 5,103) = 577.73, *p* < 0.0001. It explained 18.5% of the variance in CS (*R*^2^ = 0.185, adjusted *R*^2^ = 0.184), reflecting an overall predictive power. The model was proven able to fit this data with the non-significant lack-of-fit test results (*F* = 1.427, *p* *=* 0.144).

AS and JIF were applied as predictors and showed statistical significance: AS (*β* = 0.206, SE = 0.009, *t* = 23.85, *p* < 0.001), JIF (*β* = 0.072, SE = 0.005, *t* = 14.58, *p* < 0.001), indicating that both public/media focus and journal reputation can positively predict citation performance.

## 4 Discussion

This is the first study to compare the strength of correlations between CS and AS in research articles vs. review articles. While both article types demonstrated significant correlations between CS and AS, the correlation strength was significantly stronger in the review papers than in the research papers, likely due to their broader study involvement and more comprehensive discussion of existing literature. This illustrates that journals may benefit from incorporating active social media dissemination strategies, particularly for review articles that tend to generate higher public engagement and citation performance.

This is also the first study to individually analyze 30 top-ranked journals in sport and exercise science. We found extensive significant correlations in most of the included journals (25 out of 30 journals), suggesting a widespread association between social media attention and citation performance in sport and exercise science top-ranked journals. When the JIF is higher, the citation performance can be accordingly higher. Additionally, journals with higher JIF tended to exhibit stronger citation performance. Our results highlighted the importance and potential benefits of social media on scientific studies.

Notably, despite the fact that JIF is a well-known surrogate factor of research quality, updated evidence showed that the relationship between JIF and CS is decreasing, due to the development of the era of digitalization (15). Meanwhile, the increased number of open-access journals and online-only journals, and the easier access to pre-print publications have been changing the dissemination of research and citation performance (16–18). Our regression results supported this previously tested trend that the influence of JIF on citation performance is decreasing, while the impact of social media is increasing. In our model, AS demonstrated a stronger predictive value (*β* = 0.2060, *p* < 0.001) compared to JIF (*β* = 0.0720, *p* < 0.001), suggesting that in sport and exercise science, public/media engagement and attention may play a more substantial role in boosting citation performance than journal prestige. However, the overall explanatory power of the model was modest (*R*² = 0.185), indicating that the overall predictive power remains moderate.

As the regression model used log-transformed values, the β coefficients describe proportional changes rather than raw-unit increases in citation counts. A one-unit increase in log(AS + 1) corresponds to approximately a multiplicative increase in AS on the original scale. Back-transforming by exponentiation could convert this change into an approximate percent increase in expected CS. Therefore, the coefficients should be interpreted as reflecting the relative, rather than absolute, influence of AS and JIF on citation performance.

Despite that many CS-AS correlations were small in magnitude, such values are common in scientometric and behavioral research, where outcomes (e.g., citation counts) are influenced by contextual factors (19). A small correlation (*ρ* = 0.20–0.30) still reflects a consistent tendency for articles with higher Altmetric attention to receive more citations over time. Moderate correlations (*ρ* = 0.40–0.70), particularly those observed in several journals and in review articles, represent a more meaningful alignment between public/media engagement and scholarly impact. These effect sizes indicate that Altmetric attention captures meaningful variation in citation performance, supporting its utility as an early indicator of emerging article influence.

Scientists work diligently to achieve progress and publish their works in prestigious journals. However, these discoveries often only spread among scientific circles, remaining inaccessible to the general public. Currently, scientists in the sport and exercise science field lack experience in communicating in mainstream media. Many scientists tend to consider their contribution to mainstream media to be redundant, given that it is dominated by journalists, blog owners, fitness influencers, and podcasters (20). On the other hand, the time-consuming science work, including literature reading, experiments, and publishing, results in limited time and efforts for scientists to contribute to spreading knowledge to the general public (21).

When science outreach is led by individuals with less professional expertise, such as influencers, the accuracy and clarity of science can be compromised, leading to misinformation or confusion in the general public on social media (22), especially when people tend to rely more on social media, such as during and after the COVID-19 pandemic. The important influence of social media has been fully proven during this time. An increased number of people in the general population started to use social media to seek information in the health field. Meanwhile, numerous media with misinformation have entered the general view, causing misleading and negative medical consequences (23). While the science outreach by professionals can help addressing the pressing need of broadening health communication, our findings suggested that such efforts could associate with increased citation performance and therefore higher academic influence, which could, in turn, foster the growth of scientists and encourage more frequent sharing on social media, creating a positive and sustainable feedback loop (24).

Prestigious journals such as the *British Journal of Sports Medicine* (25) and the *International Journal of Sports Physiology and Performance* (26) have realized the importance of social media. The posts on social media can be viewed by the followers and can usually be forwarded by influencers with a larger number of followers. Therefore, social media can create a cascading information flow to sharply increase the views among the general public. By using Facebook and X, the consensus on sport-related concussion (27) has reached over 30,000 views, boosting the understanding and application for the general public and team professionals. On the other hand, as younger generations (28) who were born during and after the spread of digital technology will not distinguish between traditional media and social media, the impact of social media on research impact should not be neglected.

Several limitations must be noted in this study. Foremost, this study utilized a cross-sectional design. Because of the nature of a cross-sectional study, no causal relationship can be determined. As we included only studies published throughout the whole year of 2022, the article samples might be limited. One example is the sharp decline of articles in the *Journal of the International Society of Sports Nutrition*. The output number, including research and review articles throughout 2022, was 38, lower than the usual 50–60 volumes in other recent years. We assume the change in publisher (2021) (29) can be one of the main reasons. It could result in shifts in editorial policy with more selective acceptance criteria. Similar cases occur in *Performance Enhancement & Health* (21 articles in 2022), *Journal of Exercise Science & Fitness* (68 articles in 2022), and *Journal of ISAKOS* (40 articles in 2022). *Journal of ISAKOS* moved to a gold open-access model as of January 2022, which was considered one of the main reasons for the publication drop. While the volumes of *Performance Enhancement & Health* and *Journal of Exercise Science & Fitness* were considered normal and aligned with their deliberate editorial strategies.

Second, some omitted variables need to be noted. The research topic, study design, collaborative networks, author reputation, and funding status were not considered as predictors in the current study. Although they were all topics under sport and exercise science, the subfields can be diverse. Meanwhile, citation behavior can be shaped by collaboration networks, as multi-center or internationally coauthored papers tend to receive greater visibility and downstream citations. Topic popularity and research trends may also independently boost both AS and CS. Certain periods, such as the COVID-19 pandemic, have produced policy-driven or event-driven spikes in citations, which may not reflect typical dissemination patterns. Open-access status also affects visibility, as freely accessible articles generally accumulate more views, media attention, and citations. Some journal-led or institution-led media campaigns, or press releases, could amplify early Altmetric activity. The Altmetric platform algorithm depends on some dominant social media platforms, such as X and Facebook. In the meantime, some non-Western or non-English media platforms can be neglected. Lastly, while the *R*² value indicated a moderate effect size, we opted for a multiple linear regression model to prioritize interpretability. Future work may consider more complex machine learning models (e.g., random forests) to improve predictive accuracy.

## 5 Conclusions

In 30 top-ranked journals in sport and exercise science, AS was significantly correlated with CS, with higher strength in review articles than in research articles. Significant correlations persisted in most (25 out of 30) included journals. AS and JIF can both predict the citation performance, with AS showing higher predictive strength than JIF. These findings highlighted the importance of public/media attention in predicting academic impact. Altmetric outreach could serve as an early indicator of future success in citation performance, as a moderate and meaningful predictor.

## Data Availability

The original contributions presented in the study are included in the article/Supplementary Material, further inquiries can be directed to the corresponding author.
